# Protein responses in kenaf plants exposed to drought conditions determined using iTRAQ technology

**DOI:** 10.1002/2211-5463.12507

**Published:** 2018-09-05

**Authors:** Xia An, Guanrong Jin, Jingyu Zhang, Xiahong Luo, Changli Chen, Wenlue Li, GuangYing Ma, Liang Jin, Lunjin Dai, Xiaohua Shi, Wei Wei, Guanlin Zhu

**Affiliations:** ^1^ Zhejiang Xiaoshan Institute of Cotton & Bast Fiber Crops Flower Research and Development Centre Zhejiang Academy of Agricultural Sciences Hangzhou China; ^2^ Key Laboratory of Crop Ecophysiology and Farming Systems in the Middle Reaches of the Yangtze River Ministry of Agriculture Wuhan China; ^3^ College of Plant Science and Technology Huazhong Agricultural University Wuhan China

**Keywords:** comparative proteomic analysis, drought stress, iTRAQ, kenaf, stress resistance

## Abstract

The molecular mechanisms that underlie drought stress responses in kenaf, an important crop for the production of natural fibers, are poorly understood. To address this issue, we describe here the first iTRAQ‐based comparative proteomic analysis of kenaf seedlings. Plants were divided into the following three treatment groups: Group A, watered normally (control); Group B, not watered for 6 days (drought treatment); and Group C, not watered for 5 days and then rewatered for 1 day (recovery treatment). A total of 5014 proteins were detected, including 4932 (i.e., 98.36%) that were matched to known proteins in a BLAST search. We detected 218, 107, and 348 proteins that were upregulated in Group B compared with Group A, Group C compared with Group A, and Group B compared with Group C, respectively. Additionally, 306, 145, and 231 downregulated proteins were detected during the same comparisons. Seventy differentially expressed proteins were analyzed and classified into 10 categories: photosynthesis, sulfur metabolism, amino sugar and nucleotide sugar metabolism, oxidative phosphorylation, ribosome, fatty acid elongation, thiamine metabolism, tryptophan metabolism, plant–pathogen interaction, and propanoate. Kenaf adapted to stress mainly by improving the metabolism of ATP, regulating photosynthesis according to light intensity, promoting the synthesis of osmoregulators, strengthening ion transport signal transmission, and promoting metabolism and cell stability. This is the first study to examine changes in protein expression in kenaf plants exposed to drought stress. Our results identified key drought‐responsive genes and proteins and may provide useful genetic information for improving kenaf stress resistance.

**Abbreviations**
iTRAQ: isobaric tag for relative and absolute quantification
KEGG: Kyoto encyclopedia of genes and genomes

A shortage of water resources is a very serious environmental problem worldwide 1. Water is crucial for plant growth and development 2. Drought conditions negatively affect plant yield and/or quality and ultimately lead to considerable economic losses 3. An increase in the frequency and duration of drought conditions as well as rising temperatures in many areas poses a serious challenge to sustainable crop production 4. Moreover, drought stress adversely affects ecological stability 1. Thus, a major objective for modern agriculture involves the development of drought‐tolerant crops 5, 6.

Kenaf (*Hibiscus cannabinus* L.) is an industrial fiber crop in the family Malvaceae 7, with potential applications in textile and packing materials, the pulp and paper industry, biomass energy, composite media, potting material, building material, filtration material, board making, and animal feed 8. Kenaf, which is an important source of bast fiber, is grown worldwide, with the primary producers being China, India, and Thailand, followed by Bangladesh, Indonesia, and other countries. As early as 2002, the United States of America canceled China's textile quotas for bast fiber crops. According to data compiled by the Food and Agriculture Organization of the United Nations, the global demand for natural fibers has been increasing at an annual rate of 8%, with China contributing 15% of the global natural fiber production. Because it is a fast‐growing (i.e., short growth period) and high‐yielding plant that is resistant to environmental stresses, kenaf has become one of the most important economic crops for the eco‐friendly production of fiber. More importantly, kenaf is a drought‐tolerant plant species 7, meaning it may be grown on land that is unsuitable for food crops. Elucidating the changes in protein expression in kenaf plants under drought conditions is critical for characterizing the mechanism responsible for drought resistance. However, there have been relatively few studies regarding the drought‐induced mechanisms of kenaf plants, and the molecular mechanisms underlying their drought responses remain unclear.

Proteins directly affect the physiological and biochemical reactions in living organisms 9. Advances in proteomics technology have helped to clarify the molecular mechanisms regulating plant drought tolerance. Proteomic analyses, which involve studies of protein composition and activity 10, have generated considerable information regarding proteins involved in specific biological responses 11. Changes to leaf protein contents in two rice varieties exposed to drought stress and after a recovery period (i.e., irrigation) were investigated by MALDI‐MS and ESI‐Q‐TOF MS/MS 12. Sixteen proteins associated with drought stress responses were identified. Additionally, the abundance of 79 proteins exhibited significant changes in the leaves of sugar beet plants grown under drought conditions 13. Furthermore, proteins involved in the adaptation of *Arabidopsis thaliana* to drought stress were detected by 2D‐PAGE 14. Meanwhile, iTRAQ‐based comparative analyses of abiotic stress tolerance have been conducted for specific crops, including rice 15, grape 16, wheat 17, tomato 18, tobacco 19, cotton 11, maize 1, ramie 2, and hemp 20. Identifying stress‐responsive proteins is be important for cloning genes related to drought tolerance and for characterizing the physiological mechanism mediating plant drought tolerance.

In this study, we completed an iTRAQ‐based comparative analysis to study the molecular mechanisms underlying kenaf responses to drought stress. Our results provide the foundation for future investigations of the regulation of kenaf gene expression associated with adaptations to drought conditions. Our data may also be relevant for breeding new drought‐tolerant and high‐yielding kenaf varieties.

## Materials and methods

### Plant materials and stress treatments

Defang Li (Institute of Bast Fiber Crops, Chinese Academy of Agricultural Sciences) donated the kenaf (*Hibiscus cannabinus* L.) ‘H368’ analyzed in this study. Seedlings were grown in pots (8 cm tall with an upper diameter of 7 cm), each containing the same amount (i.e., weight) of red soil mixed with humus (1 : 1). All pots were incubated in a greenhouse with a 16‐h light (cool white fluorescent; 26 ± 1 °C): 8‐h dark (20 ± 1 °C) photoperiod and 65–70% relative humidity.

Plant growth rates were similar, as were the morphological characteristics. Plants that were approximately 30 cm tall were divided into three groups and treated as follows: Group A, watered normally (control treatment); Group B, not watered for 6 days (drought treatment); and Group C, not watered for 5 days and then rewatered for 1 day (recovery treatment). Plant C received 200 mL of water at 8:00 am per day. All stress experiments were conducted in a greenhouse under a 16‐/8‐h (light/dark) photoperiod and a photon flux density of 380 umol·m^−2^·s^−1^. For each treatment, we collected 3 g young leaf samples from individual pots. Samples were frozen in liquid nitrogen and stored at −80 °C until used for protein extractions.

**Figure 1:**
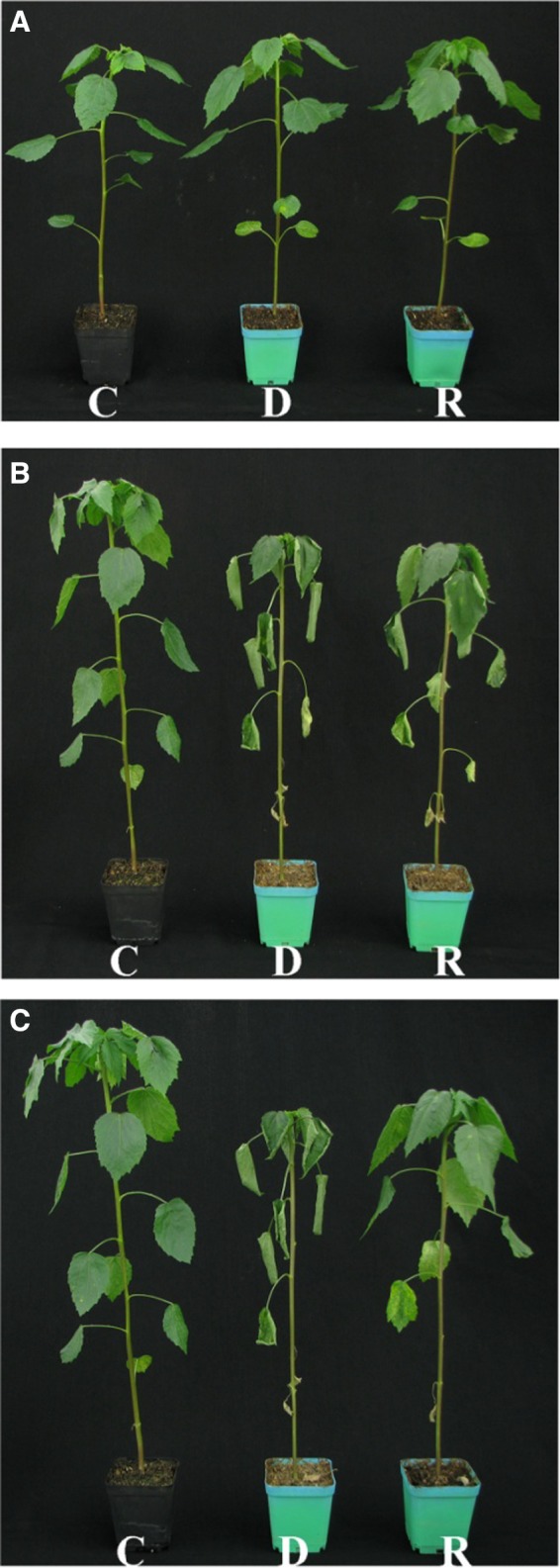
The phenotypes of kenaf seedlings were observed under normal irrigation (A), drought stress (B), and rewatering conditions (C). Plants that were approximately 30 cm tall were divided into three groups and treated as follows: Plant C represents a control plant, which was watered normally for the entire treatment period, as described in the Methods. Plant D represents a plant that was not watered for the treatment period (drought stress). Plant R represents a plant that was subjected to drought stress for 5 days and then watered on Day 6. Panel a shows the plants before treatment. Panel b shows the plants 5 days after the beginning of treatment (C was watered normally during this time, while R and D did not receive water). Panel c shows the plants 6 days after the beginning of treatment (C was watered normally during this time, D did not receive water, and R was subjected to drought stress and then watered normally on Day 6).

The physiological index (data not shown) was evaluated at eight critical time points (0, 1, 3, 5, and 6 days from the start of the drought treatment and 3, 12, and 24 h from the start of the rewatering treatment). Drought stress most directly affected plant morphology. Thus, morphological traits (Fig. 1) were screened at four critical time points (0, 5, and 6 days from the start of the drought treatment and 24 h from the start of the rewatering treatment).

### Protein extraction

Proteins were extracted from kenaf leaves as previously described, with three biological replicates for each treatment 2.

### Digestion and iTRAQ labeling

For each sample, 100 μg protein was digested overnight with trypsin (Promega, Madison, WI, USA) at 37 °C 2. Peptides were then reconstituted in 30 μL 0.5 m TEAB. Finally, two control H368 samples were labeled with isobaric tag for relative and absolute quantification (iTRAQ) tags 113 and 114. Additionally, three drought‐treated H368 samples were labeled with iTRAQ tags 115, 116, and 117, while three rewatered H368 samples were labeled with iTRAQ tags 118, 119, and 121.

### Fractionation by high‐pH reversed‐phase chromatography

The Shimadzu LC‐20AB HPLC Pump System was used for high‐pH reversed‐phase chromatography. The iTRAQ‐labeled peptide mixtures were reconstituted in 0.5 mL Buffer A (20 mm ammonium formate and 2% ACN, pH 10) and loaded onto a Gemini‐NX column (5 μm, C18, 110 Å, 250 × 4.6 mm; Phenomenex, Guangzhou, China). The peptides were eluted with Buffer A for 10 min and then with 5–30% Buffer B (20 mm ammonium formate and 98% ACN, pH 10) for 15 min. The column was then treated with 30–80% Buffer B for 3 min. All flow rates were 1 mL·min^−1^.

### LC‐ESI‐MS/MS analysis

Each fraction was resuspended in Buffer A (5% ACN and 0.1% FA) and centrifuged at 18 000 × ***g*** for 10 min. The average final peptide concentration was about 0.5 μg·μL^−1^. An 8‐μL aliquot of supernatant was loaded onto an Eksigent 425 2D HPLC System (AB Sciex, Foster City, CA, USA) at 3 μL·min^−1^ for 4 min. The peptides were eluted onto an analytical C18 column with an inner diameter of 75 μm. The column was then treated with a linear gradient of 8–25% Running Buffer B (ACN and 0.1% FA) for 45 min, a linear gradient up to 80% over 10 min, and then a 5‐min hold at 80%. The flow rate at all steps was 300 nL·min^−1^.

Data were acquired using the TripleTOF 5600 System under the following conditions: ion spray voltage, 2.3 kV; ion source gas, 6 PSI; curtain gas, 30 PSI; and interface heater temperature, 150 °C. For the MS scans, the m/z scan range was 350–1800 Da.

### iTRAQ identification and quantification of proteins

The original MS/MS data file (*.wiff) was used to search a database of transcript sequences (data not shown) using the Protein Pilot 5.0 program (AB Sciex), with the Sample Type search parameter set at iTRAQ 8‐plex (labeled peptide). Iodoacetamide was used to alkylate cysteine residues of samples that were digested with trypsin. Proteins were considered identified if the Unused ProtScore was > 1.3 (corresponding to a peptide confidence ≥ 95%). Meanwhile, the threshold used to detect significantly enriched proteins was based on a *P*‐value ≤ 0.05. A *t*‐test was applied to analyze the relative quantities of the three replicates. Up‐ or downregulated proteins were identified based on a *P*‐value < 0.05 with a twofold cutoff.

## Results and Discussion

### Protein identification and analysis

**Figure 2:**
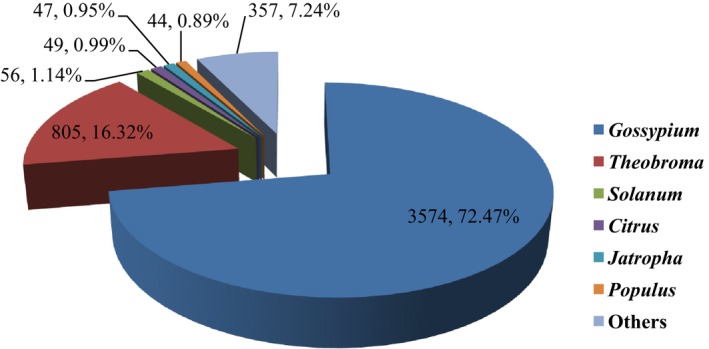
Percentage numbers of the six most abundant annotated species**.**

A total of 5014 proteins were detected, of which 4932 were matched to known sequences during a BLAST search (i.e., 98.36% of all proteins; Data S1). The top six species, which accounted for 92.76% of all matched proteins from known species, belonged to the following genera: *Gossypium* (72.47%), *Theobroma* (16.32%), *Solanum*,* Citrus*,* Jatropha*, and *Populus* (3.97%) (Fig. 2). Similar results were reported for hemp 20 and ramie 21, 22.

### Proteome‐level changes in kenaf plants exposed to drought stress

**Figure 3:**
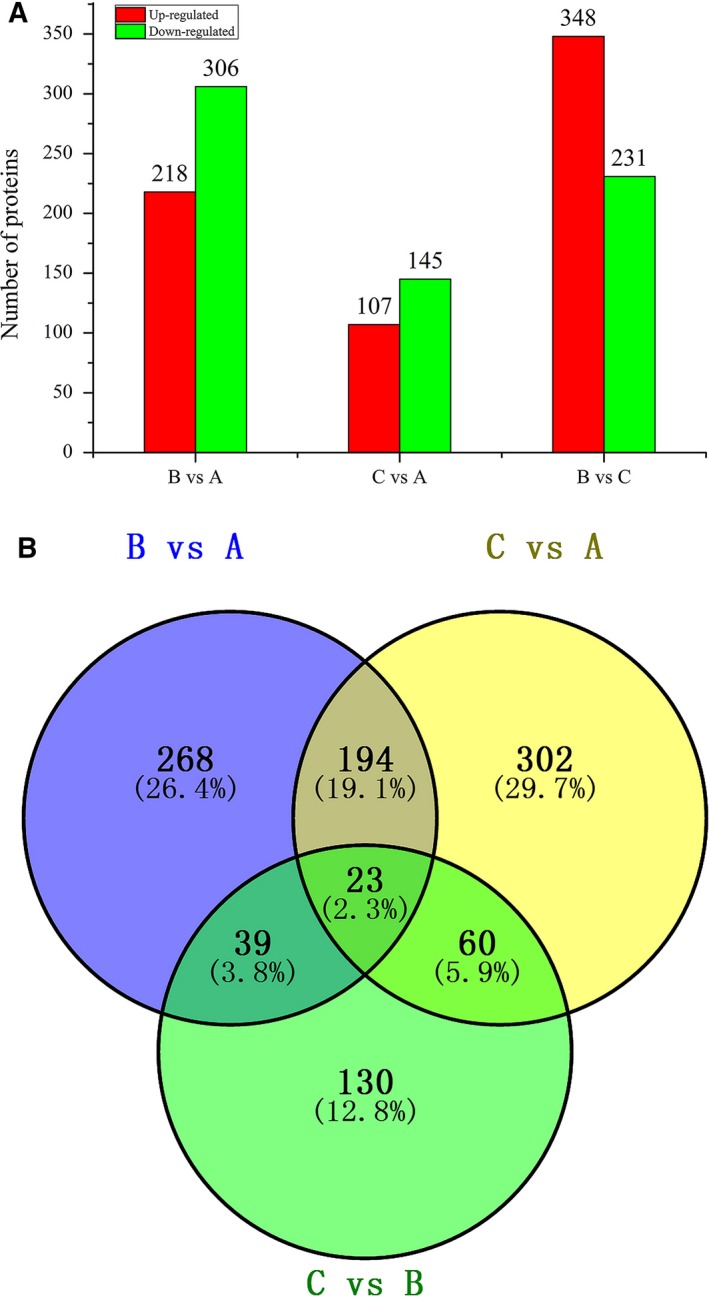
(A) The number of differentially expressed proteins in any two different groups. (B) Venn diagrams representing the overlap of identified differentially expressed proteins, which were upregulated and downregulated in any two groups.

A total of 218 and 306 proteins were up‐ and downregulated, respectively, in Group B compared with Group A. Additionally, 107 and 145 proteins were up‐ and downregulated, respectively, in Group C compared with Group A, while 348 and 231 proteins were up‐ and downregulated, respectively, in Group B compared with Group C (Fig. 3A). Our data revealed that more proteins were differentially expressed in Group B (drought treatment) than in Group C (recovery treatment). Venn diagrams of the differentially expressed proteins between any two groups are provided in Fig. 3B.

### Functional classification and annotation

**Figure 4:**
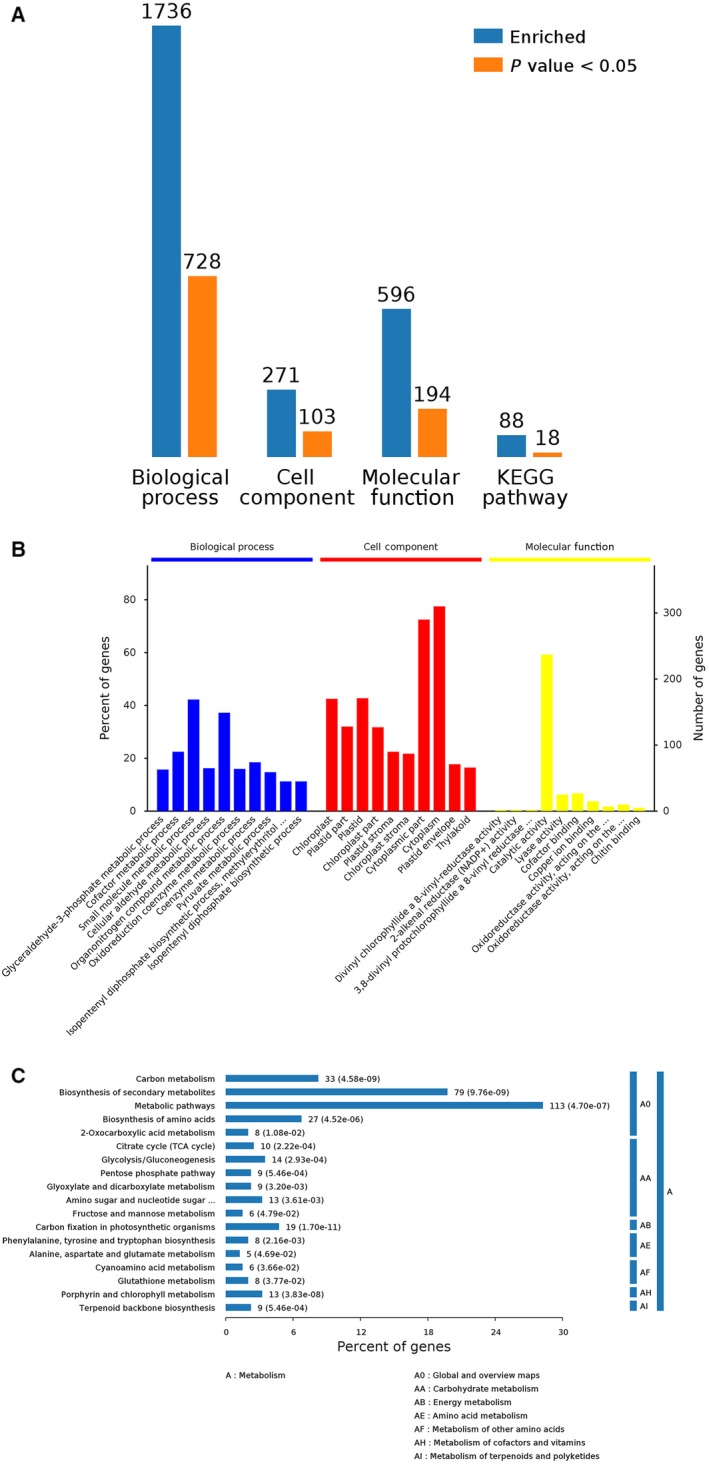
Bioinformatics analysis of 1736 identified differential abundance proteins was shown in Group B vs. Group A. Biological process, cell component, molecule function, and KEGG pathway are four categories of functional analysis (A). The ten most significantly enriched terms in level 4 GO hierarchy are shown (B). Enriched KEGG pathways are clustered into the metabolism subcategories, and the number of involved proteins in a specific pathway and corresponding *P*‐value are shown on the right side of column (C).

**Figure 5:**
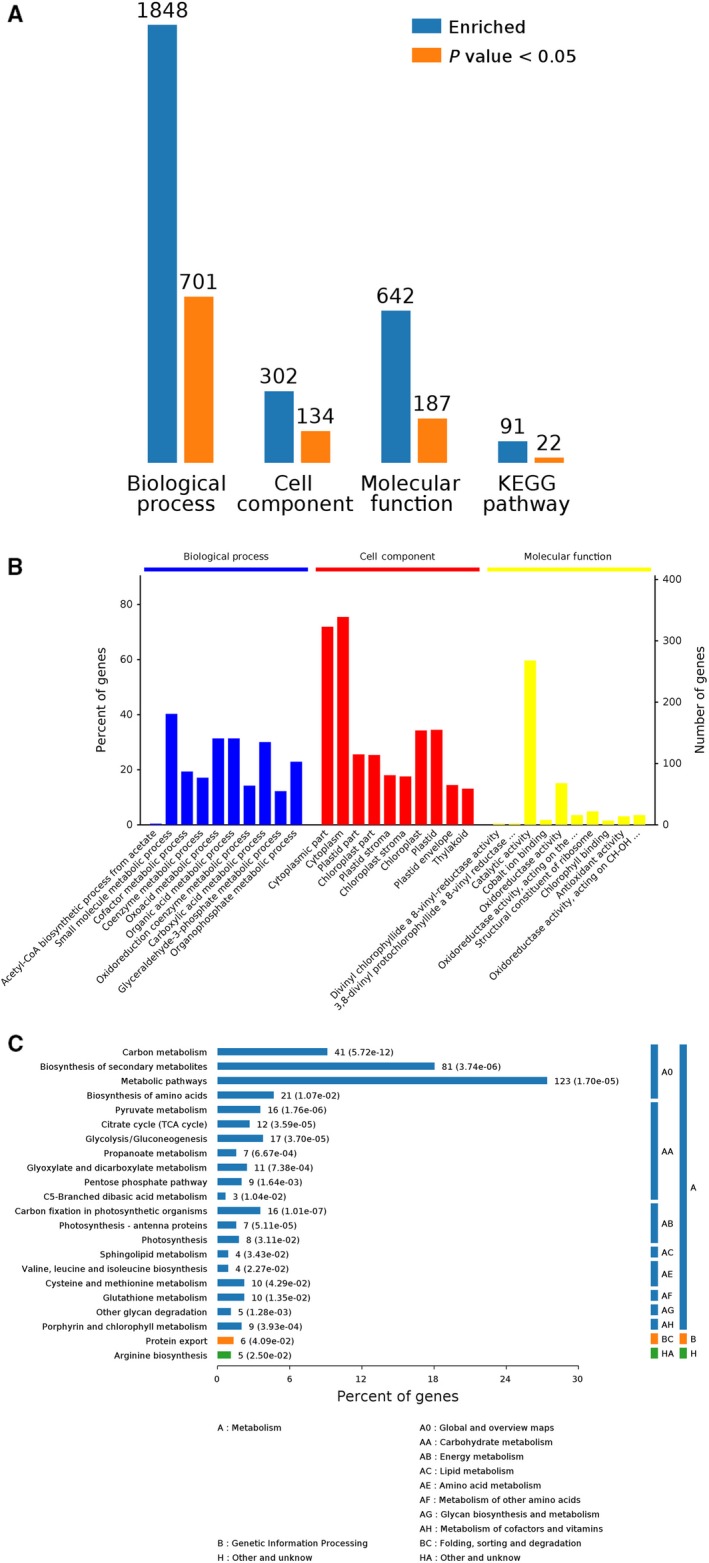
Bioinformatics analysis of 1848 identified differential abundance proteins was shown in Group C vs. Group A. Biological process, cell component, molecule function, and KEGG pathway are four categories of functional analysis (A). The ten most significantly enriched terms in level 4 GO hierarchy are shown (B). Enriched KEGG pathways are clustered into the metabolism subcategories, and the number of involved proteins in a specific pathway and corresponding *P*‐value are shown on the right side of column (C).

**Figure 6:**
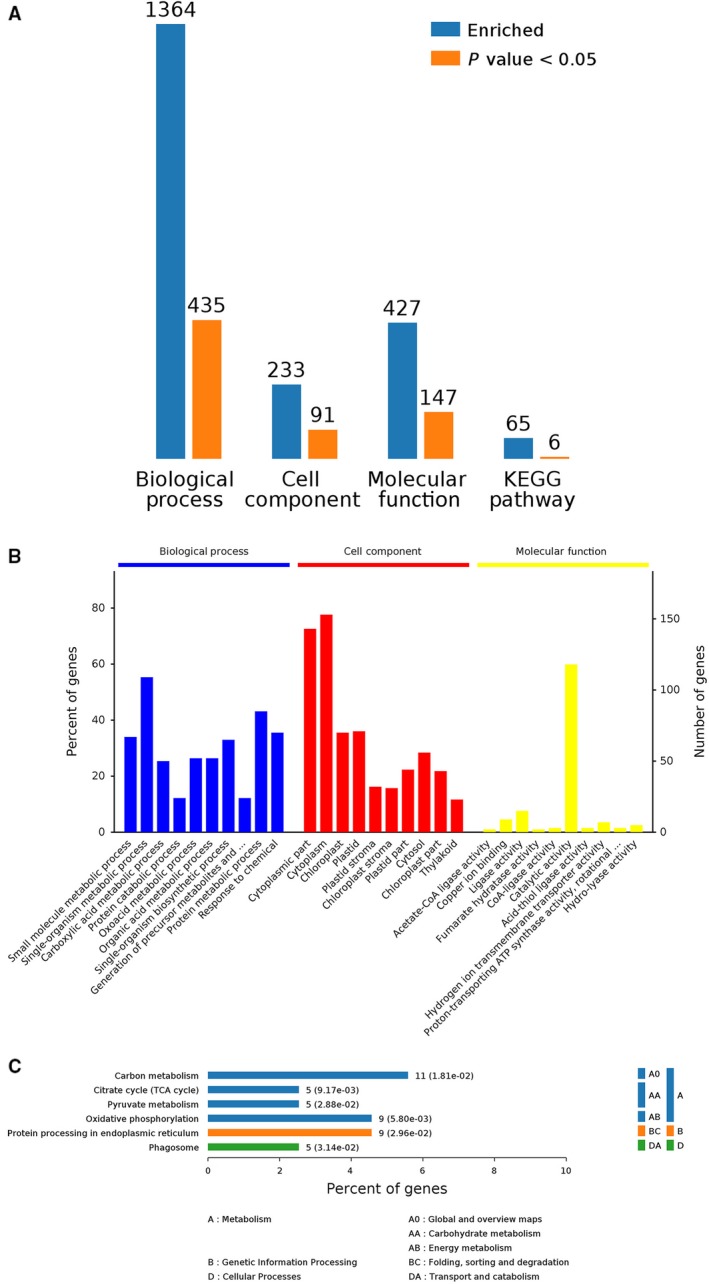
Bioinformatics analysis of 1364 identified differential abundance proteins were shown in Group C vs. Group B. Biological process, cell component, molecule function, and KEGG pathway are four categories of functional analysis (A). The ten most significantly enriched terms in level 4 GO hierarchy are shown (B). Enriched KEGG pathways are clustered into the metabolism subcategories, and the number of involved proteins in a specific pathway and corresponding *P*‐value are shown on the right side of column (C).

**Figure 7:**
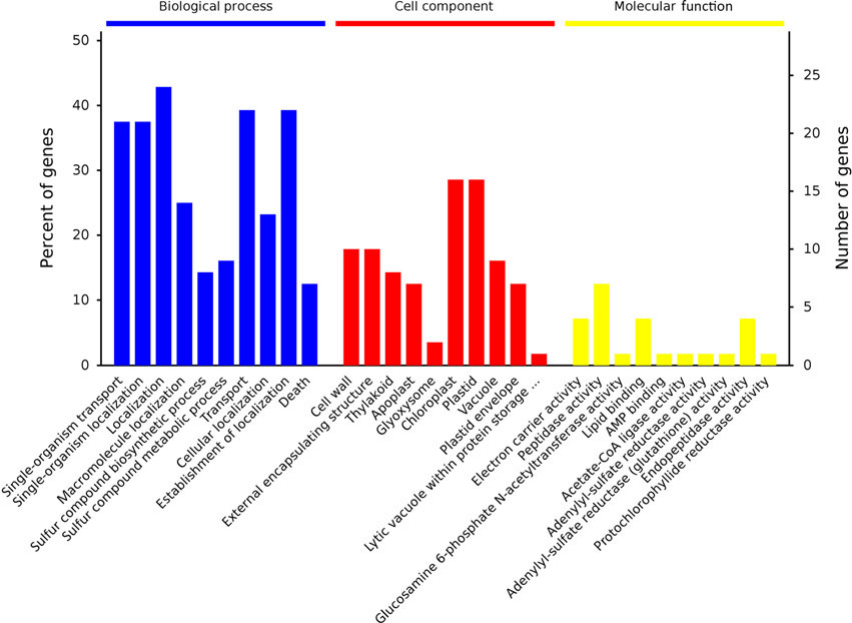
The categorization of proteins is based on GO annotation 70 identified significant differential proteins.

Proteins affected by drought conditions were functionally analyzed. All identified proteins were functionally annotated with Gene Ontology (GO) terms from the following three main categories 23: biological process (BP), cellular component (CC), and molecular function (MF). A total of 1736 proteins were annotated with a BP subcategory, and 728 types were markedly enriched (Group B vs Group A in Fig. 4A). A total of 1848 proteins were annotated with a BP subcategory, and 701 types were markedly enriched (Group C vs Group A in Fig. 5A). A total of 1364 proteins were annotated with a BP subcategory, and 435 types were markedly enriched (Group C vs Group B in Fig. 6A). Biological process, cell component, molecule function, and Kyoto encyclopedia of genes and genomes (KEGG) pathway are four categories of functional analysis. Counts for each category represent the total associated terms in the database with the query protein list. Terms with *P*‐value < 0.05 are statistically significant. Meanwhile, 271 proteins were annotated with a CC subcategory, and 103 types were markedly enriched, while 596 proteins were annotated with an MF subcategory, and 194 types were markedly enriched (Group B vs Group A in Fig. 4A). The category number is displayed with BP, cellular components, and molecular functions. *Y*‐axis (left) represents percentages of proteins identified, and *y*‐axis (right) represents the protein number. The ten most significantly enriched terms in level 4 GO hierarchy are shown in Figs 4B, 5B and 6B. The detected proteins belonged to diverse BP, CC, and MF subcategories, including 30 important functional groups (Group B vs Group A in Fig. 4B, Data S2; Group C vs Group A in Fig. 5B, Data S3; and Group C vs Group B in Fig. 6B, Data S4). ‘Small molecular metabolic processes’ represented the largest BP subcategory in Figs 5B and 6B, while ‘single‐organism metabolic process’ was the most prominent BP subcategory in Fig. 7B. In the CC category, ‘cytoplasm’ and ‘cytoplasmic part’ were the main subcategories in Figs 4B, 5B and 6B. Furthermore, ‘catalytic activity’ was the largest MF subcategory in Figs 4B, 5B and 6B. Proteins annotated with a BP subcategory single‐organism metabolic process and a molecular subcategory function catalytic activity have also been reported in ramie under drought stress, respectively 2. Excess reactive oxygen produced in plants under drought stress, if not cleared in time,can cause oxidative stress and membrane lipid peroxidation and destroy the membrane system,resulting in inhibition of plant growth. An antioxidant defense system consisting of enzymes such as SOD, POD, and CAT is formed in plants to remove excess reactive oxygen species. So the damage to plants caused by stress can be avoided.

Enriched KEGG pathways are clustered into the metabolism subcategories, the number of involved proteins in a specific pathway and corresponding *P*‐value are shown on the right side of column in Figs 4C, 5C and 6C. The KEGG analysis of Group B vs Group A (Fig. 4C; Data S2) indicated the following metabolic pathways were significantly enriched: carbohydrate metabolism, energy metabolism, amino acid metabolism, metabolism of other amino acids, metabolism of cofactors and vitamins, and metabolism of terpenoids and polyketides. Additionally, a KEGG analysis of Group C vs Group A (Fig. 5C; Data S3) revealed the following metabolic pathways were significantly enriched: carbohydrate metabolism; energy metabolism; lipid metabolism; amino acid metabolism; metabolism of other amino acids; glycan biosynthesis and metabolism; metabolism of cofactors and vitamins; and folding, sorting, and degradation. Furthermore, a KEGG analysis of Group C vs Group B (Fig. 6C; Data S4) indicated the following metabolic pathways were significantly enriched: carbohydrate metabolism; energy metabolism; folding, sorting, and degradation; and transport and catabolism.

### Gene ontology enrichment analysis for differentially expressed proteins

The expression levels of 70 differentially expressed proteins (Data S5) were examined to decrease the number of possible targets. With a fold‐change cutoff of 10 for increased accumulation and *P* < 0.1 for decreased accumulation, 17 proteins were differentially accumulated and six proteins were downregulated in Group B vs Group A. Additionally, 34 proteins were differentially accumulated and one protein was downregulated in Group C vs Group A. With a fold‐change cutoff of 4 for increased accumulation and *b* < 0.25 for decreased accumulation, nine proteins were differentially accumulated and 13 proteins were downregulated in Group C vs Group B. Genes gi|823175577 and gi|224088250 were detected in the Group B vs Group A and Group C vs Group B comparisons. Furthermore, genes gi|728833390, gi|728833158, gi|823256519, gi|823189918, gi|403391437, gi|728844267, gi|321272239, and gi|823263864 were detected in the Group B vs Group A and Group C vs Group A comparisons. The data suggested that these eight proteins play an important role in the process of kenaf drought stress treatment and rehydration treatment.

The functions of differentially expressed proteins in response to drought stress were further analyzed. And 70 proteins were grouped into BP, CC, and MF categories. Diverse BP, CC, and MF subcategories were detected, including 30 important functional groups (Fig. 7; Data S6). The predominant BP and MF subcategories were ‘localization’ and ‘peptidase activity’, respectively, while the main CC subcategories were ‘chloroplast’ and ‘plastid’.

### Regulatory changes in the pathways of 70 differentially expressed proteins

**Figure 8:**
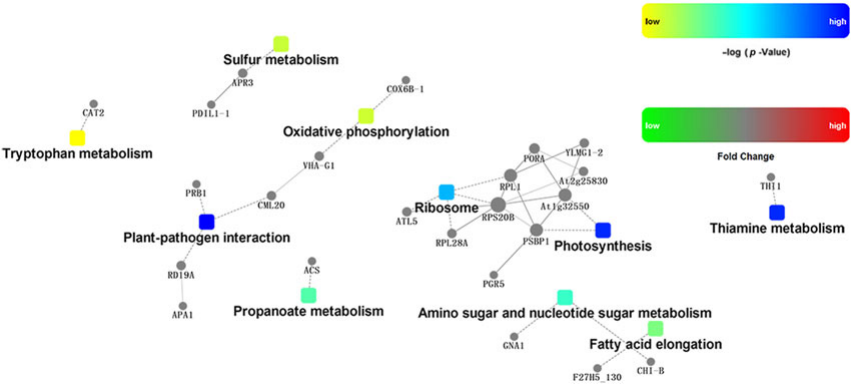
The above network model is generated with a Cytoscape web application, based on information gained from up to four levels of functional analysis: fold change of gene/protein, protein–protein interaction, KEGG pathway enrichment, and BP enrichment. Circle nodes: genes/proteins; rectangle nodes: KEGG pathway or BP. Pathways are colored in a gradient color from yellow to blue; yellow indicates a lower *P*‐value, and blue indicates a higher *P*‐value. In the case of fold‐change analysis, genes/proteins are colored in red (upregulation) and green (downregulation). A default confidence cutoff of 400 was used: Interactions with a higher confident score are shown as solid lines between genes/proteins; dashed lines indicate otherwise .

Functional analyses were conducted based on the following four factors: fold changes in gene/protein abundance, protein–protein interactions, KEGG pathway enrichment, and BP protein enrichment. A network model was generated with the Cytoscape web application (Fig. 8) 2, with up‐ and downregulated genes/proteins presented in red and green, respectively. Our results suggest that photosynthesis, sulfur metabolism, amino sugar and nucleotide sugar metabolism, and oxidative phosphorylation are vital processes associated with kenaf leaf responses to drought stress (Fig. 8). This is consistent with earlier findings that suggested H_2_‐alleviated Cd resistance is associated with activated sulfur metabolism 24, 25, 26.

### Proteins related to amino acid and protein metabolism

An effect that may occur during abiotic stress condition exposure is the participation of amino acids metabolism 27. One of the most widespread metabolic responses of plants to stresses involves the accumulation of free proline 28. An earlier study confirmed that the concentration of free proline increases under drought conditions 29. Proline functions as an osmoregulator that can decrease the damage caused by abiotic stresses 27. Free proline accumulation is one of the most widespread metabolic responses of plants to stresses. It is well known that free proline often plays a positive role in resisting drought stress. In this study, proline‐rich protein‐like isoform X6 (gi|823122770) and hybrid proline‐rich protein (gi|321272239) were upregulated by drought stress. Similar results have been reported in ramie 29. Meanwhile, 40S ribosomal protein S20‐2 (gi|747059828), 60S ribosomal protein L28‐1‐like (gi|823238069), and 50S ribosomal protein L1 (gi|702426279) were the identified proteins involved in protein translation, processing, and degradation. We observed that 50S ribosomal protein L1 and 60S ribosomal protein L28‐1‐like were downregulated, while 40S ribosomal protein S20‐2 was upregulated. Differences in the regulation of distinct components of the translation machinery suggest that a complex mechanism regulates protein synthesis in response to drought stress in kenaf 27, 30.

### Proteins related to energy metabolism

Plant growth, development, and stress responses require a considerable amount of energy in the form of ATP 31. Energy metabolism is often suppressed in plants exposed to abiotic stresses 20. Stress‐tolerant plants increase the synthesis of organic osmotic substances via enhanced energy metabolism, which is important for adaptations to abiotic stresses 20, 32. In drought‐stressed kenaf leaves, ATP‐citrate synthase alpha chain protein 3 (gi|702419922) and V‐type proton ATPase subunit G1‐like (gi|823221216) were significantly affected, with ATP‐citrate synthase alpha chain protein 3 being downregulated. The V‐type proton ATPase subunit G1‐like protein participates in the transfer of electrons from the low to high direction along the redox potential and releases energy. This process pumps protons from the mitochondrial matrix into the intercellular and outer membrane spaces, resulting in an electrochemical gradient (i.e., proton‐motive force), promotes the protons through the ATPase channel back to the substrate, and drives the synthesis of ATP 20. One of the initial plant responses to abiotic stresses involves decreasing energy metabolism by lowering the rate of ATP synthesis 2. A previous study revealed that the abundance of a protein related to ATP synthesis declines in spring wheat ‘Ningchun 4’ grown under drought conditions 33.

### Proteins related to photosynthesis

Photosynthesis is sensitive to drought and other stresses (e.g., nutrient stress) 34, 35 and is often among the first plant activities altered by stress 2. In this study, ferredoxin (gi|823213189) was downregulated under drought conditions, but then upregulated after the plants were rewatered. An analysis of illumination intensity indicated ferredoxin helps to regulate the Calvin cycle and pentose phosphate pathway. Moreover, it can balance the synthesis and degradation of sugar in plants 20, 35. Drought and strong illumination are very common environmental conditions in the main kenaf‐growing areas. Ferredoxin can regulate photosynthesis based on light intensity, thereby protecting plants from light damage under drought conditions.

## Author contributions

This article was written, guided, and amended preliminarily by XA. XA and GJ contributed reagents and materials. XA, GJ, JZ, XL, CC, WL, GM, LJ, LD, XS, WW, and GZ participated in data collection and amendment. All authors read and approved the final manuscript.

## Supporting information


**Data S1**. Proteins identified by BLAST.

**Data S2.** The detected proteins belonged to diverse BP, CC, and MF subcategories in Group B vs Group A.

**Data S3.** The detected proteins belonged to diverse BP, CC, and MF subcategories in Group C vs Group A.

**Data S4.** The detected proteins belonged to diverse BP, CC, and MF subcategories in Group C vs Group B.

**Data S5.** The expression levels of 70 differentially expressed proteins.

**Data S6**. 70 proteins were grouped into BP, CC, and MF categories.
